# Impact of corona-phobia on attitudes and acceptance towards COVID-19 vaccine among cancer patients: a single-center study

**DOI:** 10.2217/fon-2021-1015

**Published:** 2021-11-29

**Authors:** Dilek Erdem, Irem Karaman

**Affiliations:** ^1^VM Medical Park Samsun Hospital, Department of Medical Oncology, Samsun, Turkey; ^2^Medical Student(MS)/Intern Doctor, School of Medicine, Bahcesehir University, Istanbul/TURKEY

**Keywords:** acceptance, attitude, cancer, Coronavirus-19 phobia, COVID-19, hesitancy, survey, vaccine

## Abstract

**Aim:** This study aimed to assess the impact of coronavirus disease 2019 (COVID-19) phobia and related factors on attitude towards COVID-19 vaccine in cancer patients. **Methods:** A prospective cross-sectional descriptive study was conducted with 300 adult patients using a validated COVID-19 Phobia Scale (C19P-S) and related survey to determine the factors affecting vaccine acceptance between May–June 2021. **Results:** Regarding the COVID-19 vaccine willingness, 86.7% accepted vaccination, 6.3% were hesitant and 7% refused vaccination. Patients that accepted vaccination had significantly higher C19P-S scores in general, and in psychological and psychosomatic subdivisions. Univariate analysis revealed that increased age, being retired, and being married were significantly associated with willingness to be vaccinated against COVID-19. **Conclusion:** The majority of patients had high coronophobia levels which were associated with increased willingness for the COVID-19 vaccines. Minimizing negative attitudes towards vaccines will most likely be achieved by raising awareness in the cancer population about COVID-19 vaccine.

## Lay abstract

Cancer patients are considered among the privileged group for the coronavirus disease 2019 (COVID-19) vaccination. The investigators conducted a survey assessing the relationship between coronaphobia and related factors and vaccine acceptance among patients with cancer. A total of 300 patients completed a questionnaire assessing the factors affecting vaccine acceptance. Most (86.7%) patients accepted vaccination and have higher levels of fear against COVID-19, while 7% refused vaccination. Patients who have increased age, are retired and married had a higher tendency to accept vaccination against COVID-19. The main reason for acceptance was the willingness to get protection, whereas the main reason for refusal was the fear of adverse effects.

## Introduction

The severe acute respiratory syndrome coronavirus 2 (SARS-CoV-2) pandemic, which is widely known as “COVID-19”, has been a great concern for more than 5.5 million over 144 countries [1]. More than 190 million people have been infected worldwide by July 19 2020, and more than 4 million people died [2]. Although SARS-CoV-2 can cause a spectrum of diseases that are ranging from mild respiratory disease to severe pneumonia, COVID-19 possess a particular risk among the higher-risk population, such as people who have cancer, chronic kidney/heart diseases, and immunocompromised state [3].

Along with being an important threat to human life, the pandemic has led to unexpected public health problems by disrupting health care systems, economies, and mental health of the public health population [4]. To curb the spread of this novel virus, World Health Organization suggested mask-wearing and social distancing as major preventive behaviors at the beginning of 2020. However, it was suggested that long-term control of the COVID-19 pandemic relied on the development and uptake of an effective vaccine [5]. Several different pharmaceutical companies have urgently begun to develop an effective vaccine. Currently, in Turkey, emergency use approval was given for two different types of SARS-CoV-2 vaccine (Pfizer-BioNTech and CoronaVac/Sinovac) after January 2020 [6]. Omer *et al.* reported that the threshold for herd immunity should be between 50% and 67% to provide community protection [7]. Additionally, a central aspect of the efficiency of vaccines is the willingness of the population to get vaccinated [8]. Vaccination has always raised doubts in human history, furthermore, vaccine hesitancy was named as one of the top ten threats to global health in 2019 by WHO [9]. The reasons for refusing vaccination include concerns about the safety and efficacy of vaccines and the fear of the adverse effects. In particular, the development of new SARS-CoV-2 vaccines in a relatively short time with the help of novel techniques created many question marks among the public [10]. Although the development of a vaccine against COVID-19 was eagerly awaited by the majority of the population, vaccine hesitancy and refusal is a major problem causing a decreased trend of acceptance among the public [10].

Nonetheless, vaccinations significantly reduce morbidity and mortality, which is particularly crucial in vulnerable populations [11]. One such population is cancer patients, who are at high risk of severe complications and death due to COVID-19, caused by the presence of many risk factors such as immune suppression, advanced age, and comorbidities [11–13]. The probability of death among oncological patients with COVID-19 was estimated as 25.6% [14]. In addition, patients with cancer are one of the most fearful populations that were largely restricted their activities during the COVID-19 pandemic [15]. Therefore, analysis of the course of COVID-19 among patients with cancer, along with the impact of COVID-19 pandemic in their life, supports giving them a high priority for vaccination, particularly because seroconversion in patients with cancer infected by SARS-CoV-2 did not differ in comparison to the healthy population [15,16]. The risk of adverse effects and concerns about the safety and effectiveness are the main fears among cancer patients that are causing the vaccine hesitancy [3].

COVID-19 infection affects the psychology of society negatively and may harm their mental health. One example of the negative psychological effect of COVID-19 is the emergence of “coronaphobia”, which is defined as the excessive fear of COVID-19 [17]. Phobia is classified as an anxiety disorder, which may lead to abnormal behaviors due to excessive fear. Generally, the usage of non-scientifically proven sources of information often leads patients to develop wrong perceptions and erroneous beliefs. Moreover, the lack of data concerning the safety and efficacy of the COVID-19 vaccine in cancer patients, who were excluded from initial clinical trials raised a gap of information [18]. The European Society of Medical Oncology (ESMO) released statements concerning COVID-19 vaccination in oncological patients, which supports vaccination, the monitoring of side effects, and education in patients with cancer [12]. Therefore, collecting data on the attitude of cancer patients towards the SARS-CoV-2 vaccine is fundamental in improving their perception.

Since negative attitudes towards the vaccine may have a negative influence on society's mental health by increasing the existing pandemic crisis, attitudes towards the COVID-19 vaccine should be examined. Previous studies confirmed that high coronaphobia levels increase the positive attitude towards the COVID-19 vaccine [8,19]. In order to better prepare the future vaccination campaign among patients treated or monitored for cancer, this study was conducted to examine the effect of coronophobia on the attitudes towards COVID-19 vaccine among patients with cancer.

## Material & Methods

### Survey

A prospective cross-sectional descriptive study was conducted using a questionnaire survey to detect the factors determining the vaccine acceptance and hesitancy including COVID-19 phobia of cancer patients in response to the COVID-19 pandemic (Supplementary Material). This study was performed In a community-based private hospital's Medical Oncology outpatient clinic between May–June 2021. All patients who agreed to participate in the study during this period were surveyed; none who were asked declined to participate so, accordingly, 300 patients who had appointments in May 2021 were included in this study. The questionnaire was directed to the patients who came to the outpatient clinic for follow-up purposes or treatment purposes. Patients who did not come to the outpatient clinic for their control appointment in May were interviewed by telephone survey. The survey was translated and applied in Turkish to all patients. Sufficient time was given to participants to read, comprehend and answer all the questions. Patients who previously had been vaccinated against COVID-19 were excluded from the study. In addition, patients with active psychiatric disorders, cognitive impairments or dementia, and patients who were unwilling or unable to complete the questionnaire or give consent to participate were excluded from the study.

Overall, the questionnaire was developed based on literature research. The questionnaire consisted of three parts including demographic descriptive information, COVID-19 phobia scale, and questions regarding attitudes towards the COVID-19 vaccine. The demographic characteristics of the patients (age, gender, marriage status, educational status, working status) were asked and recorded. The classification and stage of cancer diagnosis have been taken from the patient records. The COVID-19 phobia scale (C19P-S) was developed by Arpaci *et al.* [20] C19P-S consist of twenty items which are diverted into four parts including psychological factors (items 1, 5, 9, 13, 17, 20), psychosomatic factors (items 2, 6, 10, 14, 18), economic factors (items 3, 7, 11, 15, 19), social factors (items 4, 8, 12, 16). High scores mean high sub-dimension and general coronaphobia levels. Cronbach's alpha value of the C19P-S was calculated as 0.92 [20]. In the present study, Cronbach's alpha value was found as 0.83. The patients were then asked five questions to determine the factors affecting the desire to get the COVID-19 vaccine. To assess COVID-19 vaccine hesitancy rates, participants were divided into three groups: a vaccine-acceptant group (ie, willing to be vaccinated immediately), a vaccine-hesitant group (ie, prefer to wait, not determined yet), and a vaccine-refusing group.

### Ethical considerations

Confidentiality of the study participants' information was maintained throughout the study by making the participants' information anonymous and asking the participants to provide honest answers. Informed consent was obtained from each participant prior to participation. The study was performed in accordance with the Helsinki Declaration and approval for this study procedure was obtained from the Turkish Ministry of Health and Institutional Review Board of the Istinye University Faculty of Medicine (2021/05).

### Statistical analysis

NCSS (Number Cruncher Statistical System) program was used for statistical analysis. Descriptive statistical methods (mean, standard deviation, median, frequency, percentage, minimum, maximum) were used while evaluating the study data. The conformity of the quantitative data to the normal distribution was tested with the Shapiro-Wilk test and graphical examinations. Student's t-test was used for comparisons between two groups of normally distributed quantitative variables, and the Mann-Whitney U test was used for comparisons between two groups of non-normally distributed quantitative variables. One-way analysis of variance and binary evaluations with Bonferroni correction were used for comparisons between groups of more than two normally distributed quantitative variables. Kruskal-Wallis test and Dunn-Bonferroni test were used for comparisons between groups of more than two quantitative variables that did not show normal distribution. Pearson chi-square test was used to compare qualitative data. Statistical significance was accepted as p < 0.05. The evaluation of Cronbach's Alpha coefficient is made according to the following criteria: 0.0–0.40: unreliable, 0.40–0.60: low-confidence, 0.60–0.80: quite reliable, 0.80–1.00: highly reliable.

## Results

### Demographic characteristics

The study was conducted between May and June 2021 in VM Medicalpark Samsun Hospital with a total of 300 cases, 65% (n = 195) female and 35% (n = 105) male. The ages of the subjects participating in the study ranged from 22 to 86, with a mean age of 55 years. Distribution of demographic characteristics was observed in Table 1. Among the 300 oncology patients included in the survey, the relative majority were between 55–64 years old (%30.3), with the education level was less than high school degree (%49.7), and most of the participants were married (%87.3) and either not currently working or retired (%75.3) (Table 2). Primary diagnosis of patients included breast cancer (n = 105), colon cancer (n = 50), lung cancer (n = 37), ovarian cancer (n = 23), gastric cancer (n = 14) and 71 patients had cancer from different origins than listed above. While the majority of patients had advanced disease (stage 3 and 4) (69%), most of them (47.3%) were actively receiving their treatment.

**Table 1.** The distribution of demographic characteristics.

Age	Min–max (median)	22–86 (56)
	Average ± SD	55.16 ± 12.91
	<45	59 (19.7)
	45–54	74 (24.7)
	55–64	91 (30.3)
	≥65	76 (25.3)
Gender	Female	195 (65.0)
	Male	105 (35.0)
Education level	Less than high school	149 (49.7)
	High school	64 (21.3)
	Higher education	87 (29.0)
Marital status	Married	262 (87.3)
	Single	38 (12.7)
Working status	Working	74 (24.7)
	Retired	91 (30.3)
	Not working	135 (45.0)
Cancer type	Breast	105
	Colon	50
	Lung	37
	Ovarian	23
	Gastric	14
	Others	71
Cancer stage	Stage I	15 (5.0)
	Stage II	78 (26.0)
	Stage III	90 (30.0)
	Stage IV	117 (39.0)
Treatment status	Control appointment	158 (52.7)
	Active treatment	142 (47.3)
Comorbid chronic illnesses	No	178 (59.3)
	Yes	122 (40.7)
	1 disease	83 (68.0)
	≥2 disease	39 (32.0)

**Table 2.** Distribution of demographic characteristics related with COVID-19.

Demographic characteristics		n (%)
Relative/friend lost due to COVID-19	No	230 (76.7)
	Yes	70 (23.3)
Did you get Influenza vaccine this year?	Yes	45 (15.0)
	No	255 (85.0)
Reason for not getting Influenza vaccine (n = 255)	Could not reach the vaccine	41 (16.1)
	Did not want to get vaccinated	214 (83.9)
Willingness to get COVID-19 vaccine	As soon as it arrives	260 (86.7)
	Doubtful	19 (6.3)
	Refusing to get vaccinated	21 (7.0)
Reason for COVID-19 vaccine acceptance (n = 260)	COVID-19 fear	31 (11.9)
	Willingness to get protection	147 (56.5)
	Duty consciousness for public	40 (15.4)
	Willingness to go back to normal life	42 (16.2)
Reason for COVID-19 vaccine hesitancy (n = 19)	Unsure about its effectiveness	5 (26.3)
	Unsure about the safety	6 (31.6)
	Unsure about which COVID-19 vaccine to take	8 (42.1)
Reason for COVID-19 vaccine refusal (n = 21)	Lack of scientific studies	7 (33.3)
	Fear of adverse effects	10 (47.7)
	The idea of COVID-19 is a benign disease	4 (19.0)
Do you trust your oncologist?	Yes	300
	No	0
Are you going to act in the same direction of your oncologist's recommendations?	Yes	300
	No	0

Among patients with chronic comorbid illnesses (40.7%), almost two-third of patients had one comorbid disease (68%), and 32% of patients had two morbid diseases including diabetes mellitus, hypertension, hyperlipidemia and chronic heart diseases.

Demographic characteristics related to COVID-19 pandemic and vaccines were presented in Table 2. None of the patients had transmitted COVID-19 during pandemic. Almost one-fourth (23.3%) of the patients lost their relative due to COVID-19. While it was observed that 15% (n = 45) of the cases were vaccinated against Influenza virus during COVID-19 pandemic, 85% (n = 255) were not vaccinated. 16.1% (n = 41) of the unvaccinated patients stated that they were not vaccinated because they could not reach the vaccine due to shortage, and 83.9% (n = 214) of the patients stated that they did not want to be vaccinated.

Regarding the COVID-19 vaccine willingness, 86.7% (n = 260) of the patients stated that they will receive the vaccine as soon as it arrived, 6.3% (n = 19) stated that they are hesitant and 7% (n = 21) stated that they will not be vaccinated. Among patients who are willing to be vaccinated, the majority of them (56.5%) accepted vaccination due to its protective effect, 16.2% (n = 42) patients stated the reason for vaccination was their desire to return to normal life, and 15.4% (n = 40) patients stated that they felt vaccination as their sense of duty for the society. and 11.9% (n = 31) of the patients would be vaccinated because of their fear of transmission of COVID-19. Of those who were doubtful about getting vaccinated, 31.6% (n = 6) were worried about its safety, 26.3% (n = 5) were unsure about its effectiveness due to lack of information regarding whether the vaccine would work or not, and 42.1% (n = 8) stated that they were unsure since they did not know which vaccine should be chosen. The majority of patients (47.7%) who refused to be vaccinated were afraid of its adverse effects, and others either found the scientific studies as insufficient (33.3%) or thought that COVID-19 was a benign disease (19%). All patients declared that they trusted their oncologist's recommendation, and they will act in the same direction as their oncologists' advice.

### Distribution of answers in COVID-19 phobia scale

The distribution of the answers given by the subjects participating in the study to the COVID-19 phobia scale was shown in Supplementary Table 1. The distribution of scores on the C19P-S and evaluation of its internal consistency was presented in Table 3. The C19P-S consisted of 20 questions with four parts including psychological, psychosomatic, economic, and social factors. The overall consistency of the scale was found highly reliable (0,836) according to Cronbach's Alpha coefficient. It was found that the participants' psychological sub-dimension mean score of C19P-S scale was 23,19 ± 5,42, while psychosomatic sub-dimension mean score was 7,27 ± 2,63 social sub-dimension mean score was 6,60 ± 3,11, economic sub-dimension mean score was 20,04 ± 3,69 and total scale mean score was 57,11 ± 11,40.

**Table 3.** Distribution of scores in the C19P-S and evaluation of internal consistency.

COVID-19 phobia scale	Number of items	Min–max (median)	Average ± SD	Cronbach's Alpha
Psychological factors	6	6–30 (24)	23.19 ± 5.42	0.749
Psychosomatic factors	5	5–25 (7)	7.27 ± 2.63	0.593
Economic factors	4	6–25 (21)	20.04 ± 3.69	0.688
Social factors	5	4–18 (5)	6.60 ± 3.11	0.581
Total score	20	21–90 (57.50)	57.11 ± 11.40	0.836

SD: Standard deviation.

### Comparisons of C19P-S scores according to demographical characteristics

The comparisons of C19P-S scores according to age groups, gender, educational level, marriage status, working status, and the number of comorbid illnesses were shown in Table 4. No significant difference was found between the scores of the patients regarding age, educational level, working status. The scores of the women in the psychological, psychosomatic, and economic sub-dimensions of the C19P-S and the total scale were statistically significantly higher than men (p = 0.012; p = 0.011; p = 0.009; p = 0.002; p < 0.05). Single patients had significantly higher psychosomatic scores in the C19P-S than the married patients (p = 0.15; p < 0.05). According to the results of the pairwise comparison made to determine the difference, patients who had two or more chronic diseases had significantly higher scores in the psychological factors sub-dimension of the C19P-S and in the total scores of the C19-PS than patients who had one chronic disease and patients who have no chronic diseases (p < 0.01).

**Table 4.** The comparisons of C19P-S scores according to different demographical parameters.

Demographical parameters			Psychological factors	Psychosomatic factors	Economic factors	Social factors	Total score
Age	<45 (n = 59)	Min–max (median)	23 (6–30)	7 (5–17)	21 (6–25)	6 (4–17)	57 (21–85)
		Average ± SD	22.61 ± 6.26	7.44 ± 2.77	19.46 ± 3.94	6.93 ± 3.27	56.44 ± 13.09
	45–54 (n = 74)	Min–max (median)	25.5 (10–30)	7 (5–15)	21 (12–25)	6 (4–16)	59.5 (35–82)
		Average ± SD	23.89 ± 4.9	7.28 ± 2.31	20.84 ± 3.24	6.8 ± 3.03	58.81 ± 10.6
	55–64 (n = 91)	Min–max (median)	23 (8–30)	7 (5–16)	21 (7–25)	6 (4–18)	57 (29–79)
		Average ± SD	23.1 ± 4.54	7 ± 1.99	19.92 ± 3.82	6.47 ± 2.86	56.49 ± 9.69
	≥65 (n = 76)	Min–max (median)	25 (7–30)	7 (5–25)	21 (11–25)	4.5 (4–18)	57 (30–90)
		Average ± SD	23.09 ± 6.15	7.45 ± 3.42	19.87 ± 3.7	6.32 ± 3.38	56.72 ± 12.65
		p	0.612^†^	0.930^†^	0.208^†^	0.360^†^	0.534^‡^
Gender	Female (n = 195)	Min–max (median)	25 (6–30)	7 (5–25)	21 (7–25)	6 (4–18)	59 (28–90)
		Average ± SD	23.84 ± 5.01	7.51 ± 2.74	20.3 ± 3.71	6.94 ± 3.27	58.58 ± 11.14
	Male (n = 105)	Min–max (median)	23 (6–30)	7 (5–21)	21 (6–25)	4 (4–13)	55 (21–84)
		Average ± SD	22.01 ± 5.95	6.83 ± 2.4	19.57 ± 3.63	5.98 ± 2.72	54.39 ± 11.44
		p	0.012^§^^,^^#^	0.011^§^^,^^#^	0.058^§^	0.009^§^^,^^††^	0.002^¶^^,^^††^
Education	Less than high school (n = 149)	Min–max (median)	24 (7–30)	7 (5–25)	21 (7–25)	6 (4–18)	58 (29–90)
		Average ± SD	23.37 ± 5.26	7.36 ± 2.68	20.34 ± 3.73	6.64 ± 3.12	57.7 ± 11.19
	High school (n = 64)	Min–max (median)	24 (13–30)	7 (5–21)	20 (13–25)	7 (4–15)	58 (36–84)
		Average ± SD	23.8 ± 4.81	7.66 ± 2.99	20.06 ± 3.08	7.14 ± 3.2	58.66 ± 10.64
	Higher education (n = 87)	Min–max (median)	23 (6–30)	6 (5–17)	21 (6–25)	5 (4–17)	56 (21–85)
		Average ± SD	22.46 ± 6.06	6.84 ± 2.23	19.53 ± 4	6.15 ± 3.01	54.98 ± 12.1
		p	0.491^†^	0.110^†^	0.175^†^	0.124^†^	0.091^‡^
Marital status	Married (n = 262)	Min–max (median)	24 (6–30)	7 (5–21)	21 (7–25)	5 (4–18)	57 (28–84)
		Average ± SD	23.4 ± 5.3	7.13 ± 2.46	20.15 ± 3.55	6.58 ± 3.1	57.27 ± 10.95
	Single (n = 38)	Min–max (median)	23 (6–30)	8 (5–25)	20.5 (6–25)	5.5 (4–17)	58.5 (21–90)
		Average ± SD	21.76 ± 6.06	8.21 ± 3.56	19.29 ± 4.52	6.79 ± 3.27	56.05 ± 14.25
		p	0.117^§^	0.015^§^^,^^#^	0.386^§^	0.728^§^	0.540^¶^
Working status	Currently working (n = 74)	Min–max (median)	24 (6–30)	7 (5–21)	21 (6–25)	5 (4–17)	57.5 (21–85)
		Average ± SD	22.97 ± 6.11	7.24 ± 2.73	19.76 ± 3.92	6.65 ± 3.09	56.62 ± 12.7
	Retired (n = 91)	Min–max (median)	24 (7–30)	7 (5–18)	21 (11–25)	5 (4–13)	56 (30–84)
		Average ± SD	22.54 ± 5.62	6.88 ± 2.22	20 ± 3.37	6.19 ± 2.75	55.6 ± 10.94
	Not working (n = 135)	Min–max (median)	24 (9–30)	7 (5–25)	21 (7–25)	6 (4–18)	58 (29–90)
		Average ± SD	23.76 ± 4.83	7.55 ± 2.82	20.23 ± 3.78	6.86 ± 3.35	58.4 ± 10.89
		p	0.356^¶^	0.132^†^	0.584^†^	0.324^†^	0.178^‡^
Co-morbid illnesses	None (n = 178)	Min–max (median)	23.5 (6–30)	7 (5–21)	21 (7–25)	5 (4–18)	57 (28–85)
		Average ± SD	22.84 ± 5.52	7.34 ± 2.7	19.99 ± 3.69	6.42 ± 3.07	56.6 ± 11.49
	1 Disease (n = 83)	Min–max (median)	24 (6–30)	7 (5–18)	21 (6–25)	6 (4–16)	56 (21–84)
		Average ± SD	22.69 ± 5.71	7.04 ± 2.16	19.51 ± 4.08	6.51 ± 2.97	55.73 ± 11.9
	≥2 disease (n = 39)	Min–max (median)	27 (18–30)	7 (5–25)	22 (16–25)	8 (4–18)	62 (50–90)
		Average ± SD	25.9 ± 3.21	7.44 ± 3.23	21.44 ± 2.25	7.64 ± 3.49	62.41 ± 8.24
		p	0.005^†^^,^^††^	0.797^†^	0.052^†^	0.117^†^	0.001^‡^^,^^††^

† Kruskal Wallis test.

‡ One-way ANOVA.

§ Mann Whitney U test.

¶ Student-t test.

# p < 0.05.

†† p < 0.01.

### Comparisons of C19P-S scores according to clinical characteristics

The comparison of C19P-S scores according to disease stages and treatment status of the patients was shown in Table 5. A statistically significant difference was found between the scores of the patients in the psychosomatic sub-dimension of the C19P-S according to the disease stages (p = 0.049; p < 0.05). According to the results of the pairwise comparison made to determine the difference; the scores of the patients with stage IV cancer had significantly higher scores than patients with stage II cancer (p = 0.047; p < 0.05). No significant difference in the C19-PS scores was found according to the treatment status of the patients.

**Table 5.** The comparison of C19P-S scores according to clinical characteristics.

Clinical characteristics			Psychological factors	Psychosomatic factors	Economic factors	Social factors	Total score
Stage	Stage I (n = 15)	Min–max (median)	24 (13–30)	7 (5–21)	21 (14–25)	4 (4–17)	57 (36–85)
		Average ± SD	23.53 ± 5.51	7.93 ± 4.64	20.6 ± 2.9	6.53 ± 3.91	58.6 ± 13.03
	Stage II (n = 78)	Min–max (median)	23 (8–30)	6 (5–25)	21 (10–25)	4 (4–16)	55 (35–90)
		Average ± SD	23.09 ± 5.54	6.94 ± 3.06	19.95 ± 3.49	6.08 ± 2.83	56.05 ± 11.14
	Stage III (n = 90)	Min–max (median)	25 (7–30)	7 (5–16)	21 (11–25)	6 (4–18)	58 (32–80)
		Average ± SD	23.58 ± 4.94	7.33 ± 2.34	20.34 ± 3.69	6.68 ± 3.09	57.93 ± 10.84
	Stage IV (n = 117)	Min–max (median)	24 (6–30)	7 (5–18)	21 (6–25)	6 (4–18)	58 (21–84)
		Average ± SD	22.93 ± 5.72	7.36 ± 2.19	19.8 ± 3.92	6.91 ± 3.21	57 ± 11.86
		p	0.945^†^	0.049^†^^,^^#^	0.636^†^	0.154^†^	0.706^‡^
Treatment status	Control appointment (n = 158)	Min–max (median)	24 (6–30)	7 (5–25)	21 (10–25)	6 (4–18)	59 (28–90)
		Average ± SD	23.75 ± 5.17	7.28 ± 2.99	20.04 ± 3.58	6.66 ± 3.21	57.74 ± 11.39
	Active treatment (n = 142)	Min–max (median)	24 (6–30)	7 (5–18)	21 (6–25)	5 (4–18)	57 (21–84)
		Average ± SD	22.58 ± 5.64	7.25 ± 2.19	20.04 ± 3.83	6.54 ± 3.02	56.42 ± 11.42
		p	0.065^§^	0.285^§^	0.855^§^	0.816^§^	0.316^¶^

† Kruskal Wallis test.

‡ One-way ANOVA.

§ Mann Whitney U test.

¶ Student-t test.

# p < 0.05.

†† p < 0.01.

SD: Standard deviation.

### Comparisons of C19P-S scores according to COVID-19 phobia-related characteristics

The comparison of C19P-S scores according to COVID-19 phobia-related characteristics was presented in Table 6. It was observed that patients who lost their relatives due to COVID-19 had significantly higher C19-PS scores in all subdivisions and the total score. Patients who were willing to be vaccinated had significantly higher C19P-S scores in general, and in psychological and psychosomatic subdivisions than patients who refuse to be vaccinated (p < 0.01). Patients were evaluated for their desire to get the COVID-19 vaccine by means of their descriptive characteristics in Table 7. Univariate analysis revealed that increased age, being retired and being married were significantly associated with willingness to be vaccinated against COVID-19 (p < 0.01).

**Table 6.** The association between of C19P-S scores and COVID-19 vaccine related parameters.

COVID-19 vaccine-related parameters			Psychological factors	Psychosomatic factors	Economic factors	Social factors	Total score
Did you lost a relative/friend due to COVID-19?	No (n = 230)	Min–max (median)	24 (6–30)	7 (5–25)	21 (6–25)	5 (4–18)	56 (21–90)
		Average ± SD	22.74 ± 5.49	7.01 ± 2.6	19.68 ± 3.71	6.32 ± 2.89	55.75 ± 11.13
	Yes (n = 70)	Min–max (median)	26 (6–30)	8 (5–18)	21 (11–25)	7 (4–18)	63 (28–85)
		Average ± SD	24.7 ± 4.91	8.11 ± 2.61	21.24 ± 3.37	7.54 ± 3.65	61.6 ± 11.2
		p	0.005^§^^,^^††^	0.001^§^^,^^††^	0.001^§^^,^^††^	0.020^§^^,^^#^	0.001^¶^^,^^††^
Did you get your annual flu (Influenza) vaccine?	Yes (n = 45)	Min–max (median)	24 (7–30)	7 (5–13)	21 (13–25)	5 (4–13)	57 (32–77)
		Average ± SD	23.29 ± 5.52	7.07 ± 1.74	20.53 ± 3.35	6.58 ± 3.08	57.47 ± 11.02
	No (n = 255)	Min–max (median)	24 (6–30)	7 (5–25)	21 (6–25)	5 (4–18)	58 (21–90)
		Average ± SD	23.18 ± 5.41	7.31 ± 2.77	19.96 ± 3.75	6.61 ± 3.13	57.05 ± 11.49
		p	0.854^§^	0.757^§^	0.400^§^	0.852^§^	0.822^¶^
Will you get the COVID-19 vaccine?	Yes (n = 260)	Min–max (median)	25 (6–30)	7 (5–25)	21 (6–25)	5 (4–18)	58 (21–90)
		Average ± SD	23.55 ± 5.46	7.33 ± 2.7	20.2 ± 3.58	6.62 ± 3.12	57.69 ± 11.38
	Doubtful (n = 19)	Min–max (median)	22 (17–30)	7 (5–15)	20 (12–25)	6 (4–12)	55 (41–77)
		Average ± SD	22.79 ± 4.09	7.63 ± 2.5	19.79 ± 3.94	7 ± 2.43	57.21 ± 10.3
	No (n = 21)	Min–max (median)	20 (8–28)	5 (5–11)	19 (7–25)	4 (4–18)	49 (29–71)
		Average ± SD	19.24 ± 4.54	6.19 ± 1.69	18.38 ± 4.52	6.1 ± 3.69	49.9 ± 10.62
		p	0.001^†^^,^^††^	0.041^†^^,^^#^	0.148^†^	0.175^†^	0.010^‡^^,^^#^

† Kruskal Wallis test.

‡ One-way ANOVA.

§ Mann Whitney U test.

¶ Student-t test.

# p < 0.05.

†† p < 0.01.

SD: Standard deviation.

**Table 7.** Evaluation of the association between willingness for COVID-19 vaccine and descriptive characteristics.

Characteristics		Willingness to get the COVID-19 vaccine		p
		Yes n (%)	Doubtful & no n (%)	
Age	<45	44 (74.6)	15 (25.4)	0.001^†^^,^^§^
	45–54	61 (82.4)	13 (17.6)	
	55–64	80 (87.9)	11 (12.1)	
	≥65	75 (98.7)	1 (1.3)	
Gender	Female	164 (84.1)	31 (15.9)	0.075^†^
	Male	96 (91.4)	9 (8.6)	
Educational level	Less than high school	134 (89.9)	15 (10.1)	0.250^†^
	High school	53 (82.8)	11 (17.2)	
	Higher education	73 (83.9)	14 (16.1)	
Marital status	Married	231 (88.2)	31 (11.8)	0.045^†^^,^^‡^
	Single	29 (76.3)	9 (23.7)	
Working status	Working	63 (85.1)	11 (14.9)	0.008^†^^,^^§^
	Retired	87 (95.6)	4 (4.4)	
	Not working	110 (81.5)	25 (18.5)	
Stage of the cancer	Stage I	12 (80.0)	3 (20.0)	0.715^†^
	Stage II	66 (84.6)	12 (15.4)	
	Stage III	78 (86.7)	12 (13.3)	
	Stage IV	104 (88.9)	13 (11.1)	
Treatment status	Control	141 (89.2)	17 (10.8)	0.167^†^
	Active treatment	119 (83.8)	23 (16.2)	
Number of co-morbid illnesses	None	149 (83.7)	29 (16.3)	0.131^†^
	1 Disease	74 (89.2)	9 (10.8)	
	≥2 Disease	37 (94.9)	2 (5.1)	
The presence of relative/friend lost due to COVID-19	No	197 (85.7)	33 (14.3)	0.349^†^
	Yes	63 (90.0)	7 (10.0)	
The presence of annual Influenza vaccine	Yes	43 (95.6)	2 (4.4)	0.057^†^
	No	217 (85.1)	38 (14.9)	

† Pearson Chi-Square test.

‡ p < 0.05.

§ p < 0.01.

## Discussion

Along with its negative social and economic impact on society, COVID-19 pandemic has led to important psychological problems including the disruption of the mental health of the public population. One negative consequence of the COVID-19 pandemic from a psychological perspective was the introduction of ‘coronophobia’ to the world [15]. In this study, authors investigated the impact of COVID-19 phobia on attitudes towards vaccine acceptance. At the time this survey was conducted, all cancer patients were eligible to receive a COVID-19 vaccine in Turkey. Therefore, there were no patients that wanted the vaccine but were not able to obtain it during the time period of the study. To the best of the authors' knowledge, although COVID-19 vaccine hesitancy and related factors in cancer patients were assessed with previous studies, to date, no particular study investigated the effect of coronophobia and related factors on vaccine acceptance among cancer patients. This study sheds light on the coronophobia levels and their effect on vaccine acceptance among people with a history of cancer, which is necessary to determine how to encourage COVID-19 vaccination for vulnerable patients, such as those with a history of cancer. The authors found that the majority of patients had high coronophobia levels, which was significantly associated with losing a relative due to COVID-19, being women, increased co-morbid illnesses, and increased stage of cancer. The increased willingness for a COVID-19 vaccine was significantly associated with increased coronophobia, increased age, being retired, and being married. In light of these findings, it is fundamental to develop strategies to encourage vaccination in the direction of current evidence-based guidelines of cancer societies. The question of “what are oncologists doing in terms of teaching in cancer population” remain an important question, whose answer relies on realizing the confounding factors that determine the vaccine acceptance.

### COVID-19 phobia

Compared to previous studies, in our study, the participants had a high level of mean C19P-S total score [15,17]. In terms of sub-dimensions, it was found that mean psychological and economic sub-dimension scores were high, while mean social and psychosomatic sub-dimension scores were low, similar to previous studies [8,21–23]. Previous studies demonstrated both psychological and physiological factors in different populations determining the COVID-19 phobia of an individual such as losing a relative due to COVID-19, having significant co-morbid illnesses [8,19,24,25]. Different results in studies conducted may be due to differences in populations, vaccine-related news and the dates of the studies conducted.

Kelkar *et al.* reported that empathy and phobia are among the important factors that give purpose for COVID-19 vaccination [26]. The result of this study demonstrated that the cancer patients were psychologically affected by showing exaggerated and abnormal behaviors as a result of excessive focus on the ways to protect them from the COVID-19 pandemic. Accordingly, increased C19P-S was associated with increased willingness for vaccination. Since older patients were at increased risk for severe COVID-19, and married patients had felt an increased sense of duty to protect their families, those patients were more likely to accept a vaccine for themselves.

### Factors that affect acceptance of COVID-19 vaccination

In this study, it was remarkable that half of the patients that accepted vaccination stated that they wanted to protect themselves against COVID-19, whereas the other half stated a reason either due to fear of COVID-19, desire to return to normal life or as a sense of duty for the society. It is seen that patients who were concerned about their family and society were more likely to accept a vaccine for themselves. Therefore, it is important to support vaccine acceptance in a campaign, such as “vaccinate if not for yourself, then for others” [26]. These findings confirm the concept of altruism for family and community [27]. The duty consciousness for the public, fear of COVID transmission, and willingness to return to normal life all can be grouped as an altruism index. In this manner, the balance between coronaphobia may induce the feeling of altruism, which may together promote vaccine acceptance [27].

Doubtful patients either stated concerns about safety, effectiveness, or indetermination about which vaccine to take. Similarly, patients who refused vaccination stated a concern regarding adverse effects, or insufficiency of scientific studies, whereas a minority of patients reported a thought such as COVID-19 has a benign course. Arce *et al.* found that educational level was a positive and significant predictor of COVID-19 vaccine acceptance in the United States [28]. On the other hand, they found a higher willingness to take a COVID-19 vaccine in lower and middle-income countries (LMIC), compared with the United States and Russia. They stated that vaccine acceptance in LMICs is primarily explained by an interest in personal protection against COVID-19, while concern about side effects is the most common reason for hesitancy [28]. When considering the relatively low educational status of our study participants, these findings raise concern for public susceptibility to negative perception towards COVID-19 vaccine by misinformed or nefarious agents or groups. This result shows that vaccine enthusiasm will most likely be boosted by positive impressions of safety and efficacy data of the COVID-19 vaccines. This study confirms and extends prior reports showing that the most formidable impediments to vaccine acceptance are safety concerns related to adverse effects and that allaying such concerns directly supports vaccine uptake [5].

It was remarkable that all patients who refused vaccination stated that they will act in the direction of their oncologists' recommendation. Since all patients agreed to this notion, it is seen that physician authority is respected for COVID-19 vaccination, which has been previously reported for other vaccines, such as vaccines against influenza [26]. However, it is now known that COVID-19 and Influenza can affect people differently. Since flu has been around much longer, physicians know much more about how to treat and prevent it, while research about COVID-19 is still ongoing. On the other hand, COVID-19 is more contagious and spread more rapidly than Influenza, and its clinical course differed significantly which may lead to a more severe disease course with serious complications that lead to death, with a higher mortality rate than Influenza. Altogether, this leads to less fear of Influenza and higher acceptance of Influenza vaccination when encouraged, while awareness of mortality might be a driver for COVID-19 vaccination [29]. To build trust in the health benefits of both influenza vaccination and, ultimately, SARS-CoV2 vaccines, robust educational campaigns and policy initiatives are required besides teaching by oncologists.

The psychological effects of coronaphobia will continue to affect different perspectives of life of cancer patients. Therefore, to shift patients' attitudes toward vaccine acceptance, there need to be opportunities for physicians to directly engage with their patients and the public regarding giving information about vaccine safety and efficacy, as well as supporting psychologically [26]. Since the most frequent rationales for vaccine hesitancy are consistent with the fact that the currently available vaccines were not tested on cancer patients, but inconsistent with recent recommendations of public health and cancer experts who believe the benefits of vaccination outweigh the risk, conveying current information to patients are a milestone in shifting their attitudes toward vaccines [30]. Main strategies to teach and encourage patients about COVID-19 vaccination with gaining high level of trust include giving clear, reliable, culturally appropriate and comprehensible debriefing targeting cancer patients to be addressed with each visit, and taking sufficient time to discuss and answer their each and every question. Emphasizing the risks of COVID-19 infection and the benefits of vaccination in the direction of development of current evidence by increasing the diversity of participants in COVID-19 vaccine trials may be needed [30].

### The current state of COVID-19 vaccination acceptance

In the present study, the participants were found to have a high level of positive attitude towards the COVID-19 vaccine. Previous studies beginning from 2020 reported a positive view of the people for the COVID-19 vaccine in most western countries varying between 59–75% [17,31–33]. A recent study by Mejri *et al.* reported a vaccine acceptance rate of 50.5% from Tunisia, whereas Moujaess *et al.* reported that 55%of the cancer patients inBeirut, Lebanon were ready to be vaccinated [34,35]. Nonetheless, our study that consisted of cancer patients revealed a higher COVID-19 vaccine acceptance (86.7%) than literature. As previosly discussed, higher level of acceptance and trust in our population of cancer patients can be attributed to importance of our strategies that encourage all patients to receive the COVID-19 vaccination.

It has been demonstrated that vaccination against COVID-19 significantly reduces the severity of the disease course and mortality, which is particularly important for patients with a weakened immune system to fight the virus, such as cancer patients [16]. Previous studies demonstrated that even patients with weakened immune systems can produce a strong antibody response after vaccination [16]. It can be seen that under the negative effect of the pandemic, individuals show a large number of preventive behaviors such as avoiding mass transportation, washing hands frequently, wearing masks to minimize the risk, and minimizing contact with other people [36]. Studies conducted have shown that perceived risk causing fear and anxiety increases positive attitude towards the COVID-19 vaccine [8,17,37]. Considering all of these, it is expected for individuals to show positive attitudes towards vaccines, which is today seen as the most effective preventive measure, as demonstrated in the present study. The results of the study are in parallel with the literature.

### Limitations

A few limitations should be considered when interpreting this study. Patients included in the present study were very heterogeneous in terms of their cancer diagnoses and only a small number of patients in our clinics were included due to the cross-sectional nature of the study. Patients with psychiatric disorders were excluded from the study; however, cancer-related anxiety that we have not yet noticed (especially in patients at the beginning of the diagnosis and treatment process) might be confounding some of the results. However, this study provides much-needed data to elucidate the factors associated with vaccine hesitancy in patients with a history of cancer.

## Conclusion

Here, a positive attitude towards the COVID-19 vaccine was observed as the level of coronaphobia increased. In line with these results, it can be recommended to provide psychological counseling to teach methods to cope with coronaphobia and to minimize negative attitudes towards vaccines by raising awareness in the society about the COVID-19 vaccine. The extent to which healthcare professionals are able to integrate these aspects in vaccination campaigns will determine its success and the course of the COVID-19 pandemic.

**Summary points**

- Previous studies confirmed that high coronaphobia levels increase the positive attitude towards the COVID-19 vaccine. Although COVID-19 vaccine hesitancy and related factors in cancer patients were assessed with previous studies, to date, no particular study investigated the effect of coronophobia and related factors on vaccine acceptance among cancer patients. In order to better prepare the future vaccination campaign among patients treated or monitored for cancer, this study was conducted to examine the effect of coronophobia on the attitudes towards COVID-19 vaccine among patients with cancer.
- Patients' psychological sub-dimension mean score of C19P-S scale was 23.19 ± 5.42, while psychosomatic sub-dimension mean score was 7.27 ± 2.63 social subdimension mean score was 6.60 ± 3.11, economic sub-dimension mean score was 20.04 ± 3.69 and total scale mean score was 57.11 ± 11.40.
- Regarding the COVID-19 vaccine willingness, 86.7% accepted vaccination, 6.3% were hesitant and 7% refused vaccination.
- Patients who lost their relatives due to COVID-19 had significantly higher C19-PS scores in all subdivisions and in total. Patients that accepted vaccination had significantly higher C19PS scores in general, and in psychological and psychosomatic subdivisions (p < 0.01).
- Univariate analysis revealed that increased age, being retired and being married were significantly associated with willingness to be vaccinated against COVID-19 (p < 0.01).
- Our study that consisted of cancer patients revealed a higher COVID-19 vaccine acceptance (86.7%) than the literature, and 100% patients stated that they were going to act on the recommendation of the oncologist. Low level of vaccine hesitancy and high level of trust in our population of cancer patients highlights the potential strategies to improve the vaccine acceptance in cancer patients.
- Main strategies to teach and encourage patients about COVID-19 vaccination with gaining high level of trust include giving clear, reliable, culturally appropriate and comprehensible debriefing targeting cancer patients to be addressed with each visit, and taking sufficient time to discuss and answer their each and every question.
- Emphasizing the risks of COVID-19 infection and the benefits of vaccination in the direction of development of current evidence are needed to improve the positive attitude.
